# In Vitro Anti-Inflammatory Effects of Phenolic Compounds from Moraiolo Virgin Olive Oil (MVOO) in Brain Cells via Regulating the TLR4/NLRP3 Axis

**DOI:** 10.3390/molecules24244523

**Published:** 2019-12-10

**Authors:** Agnese Taticchi, Stefania Urbani, Elisabetta Albi, Maurizio Servili, Michela Codini, Giovanna Traina, Stefania Balloni, Federica Filomena Patria, Luana Perioli, Tommaso Beccari, Carmela Conte

**Affiliations:** 1 Department of Agricultural, Food and Environmental Sciences, University of Perugia, 06126 Perugia, Italy; agnese.taticchi@unipg.it (A.T.); stefania.urbani@unipg.it (S.U.); maurizio.servili@unipg.it (M.S.); 2 Department of Pharmaceutical Sciences, University of Perugia, 06126 Perugia, Italy; elisabetta.albi@unipg.it (E.A.); michela.codini@unipg.it (M.C.); giovanna.traina@unipg.it (G.T.); stefania.balloni@libero.it (S.B.); patriafederica@gmail.com (F.F.P.); luana.perioli@unipg.it (L.P.); tommaso.beccari@unipg.it (T.B.)

**Keywords:** olive oil phenolic compounds, neuroinflammation, toll-like receptor 4 (TLR4), NOD-like receptor pyrin domain-containing 3 (NLRP3), microglia, neurons

## Abstract

Neuroinflammation is a feature of many classic neurodegenerative diseases. In the healthy brain, microglia cells are distributed throughout the brain and are constantly surveilling the central nervous system (CNS). In response to CNS injury, microglia quickly react by secreting a wide array of apoptotic molecules. Virgin olive oil (VOO) is universally recognized as a symbol of the Mediterranean diet. In the current study, using lipopolysaccharide (LPS)-stimulated BV2 microglia, the anti-inflammatory effects of VOO phenolic extracts from Moraiolo cultivar (MVOO-PE) were investigated. The results showed that low concentration of MVOO-PE prevented microglia cell death and attenuated the LPS-induced activation of toll-like receptor 4 (TLR4)/NOD-like receptor pyrin domain-containing-3 (NLRP3) signaling cascade. The levels of TLR4 and NF-kB were diminished, as well as NLRP3 inflammasome and interleukin-1β (IL-1β) production. Cyclooxygenase-2 (COX-2) isoenzyme and ionized calcium binding adaptor molecule 1 (Iba-1) inflammatory mediator were also reduced. By modulating the TLR4/NLRP3 axis, MVOO-PE pretreatment was able to significantly down-regulate the mRNA expression of inflammatory mediators and suppress the cytokine secretion. Finally, we showed protective effect of MVOO-PE in a transwell neuron–microglia co-culture system. In conclusion, these results suggest that MVOO-PE could exerts anti-inflammatory activity on brain cells and become a promising candidate for preventing several neuroinflammatory diseases.

## 1. Introduction

In the central nervous system (CNS), the complex neuronal network is maintained and supported by macro- and microglia, both considered the immune system guards in the brain [1,2]. In response to brain inflammatory insults, microglia activation becomes a key factor in the defense of neural parenchyma, providing for the maintenance of homeostasis and immune surveillance [3,4]. Microglia has different phenotypes in relation to neurodevelopment, homeostasis, in vitro conditions, aging, and neurodegenerative diseases [5]. Although, the terminology of macrophage polarization is most commonly used to establish the M1 and M2 phenotypes, the ontogeny and functional significance of microglia have not yet been characterized [6]. The switching from M1 to M2 phenotype depends on the degree, contest, and duration of the neuroinflammatory stimulus [7]. Although microglial activation is necessary and crucial for neuron survival, long-term activation results in neurotoxic consequences for the brain. Neuronal damage further activates inflammatory mechanism, causing a vicious cycle that culminates in neuronal death. To restore CNS homeostasis, microglia releases several inflammatory mediators, including cytokines, such as interleukin (IL-1β) and tumor necrosis factor (TNF-α), chemokines, nitrix oxide (NO), and reactive oxygen species (ROS).

Different stimuli can cause microglia overactivation. Lipopolysaccaride (LPS)-stimulated microglia represents a good model to study the underlying mechanisms of neurodegeneration. LPS is an endotoxin derived from the outer membrane of Gram-negative bacteria, which induces a potent inflammatory response through toll-like receptor 4 (TLR4) [8]. TLR4 is a pattern-recognition receptor (PRR) that recognizes distinct pathogen-associated molecular patterns (PAMPs), including LPS, and damage-associated molecular patterns (DAMPs) that are released by damaged tissues [9].

Glial cells also express several PRRs, such as NOD-like receptors (NLRs), which are well-known mediators of inflammasome [10]. NLR family pyrin domain-containing 3 (NLRP3) inflammasome is the best characterized [11] and it is activated in response to numerous stimuli. The binding of LPS to TLR4 induces the activation of nuclear factor-κB (NF-kB) and leads to NLRP3 assembly and overactivation. NLRP3 inflammasome complex, in turn, induces the activation of caspase-1, whose pivotal role is the cleavage of pro-IL-1β and IL-18 precursor to mature forms [12]. These events culminate with secretion of proinflammatory cytokines.

Some studies showed the relation between food rich in phenolic compound consumption and decreased risk for the development of chronic inflammatory diseases [13,14,15]. Hydroxytyrosol is the main phenolic compound present in olive oil, which exhibits antioxidant [16], anti-inflammatory [17], and chemopreventive properties [18].

The aim of this study was to investigate the anti-inflammatory potential of olive oil phenolic compounds from Moraiolo cultivar (Umbria, Italy). We used a widely used in vitro model of inflammation based on acute treatment of BV2 microglial cells with LPS, and examined intracellular signaling involved in neurodegeneration. Moreover, using a co-culture system, the mutual influence between microglia and neurons was explored.

## 2. Results

### 2.1. Phenolic Compounds from Moraiolo Olive Oil Protect Against LPS-Treated BV-2 Cells

In order to test the protective effect of the MVOO-PE against neuroinflammation, we first set out to measure the phenol composition of extracts. Results showed that total polyphenols were about 740 mg/mL. MVOO contained 737.5 mg/g of phenolic compounds, mainly as hydroxytyrosol derivates (76.4%), tyrosol derivates (17.6%), and lignans (6%). The most abundant polyphenol was the 3,4-DHPEA-EDA (49% of total). The second most represented was 3,4-DHPEA-EA (26%), followed by p-HPEA-EDA (14%). The minor components in a progressively decreasing order were (+)-1-acetossipinoresinolo (4.9%), Ligustroside Aglicone (2.4%), 3,4-DHPEA (2.1%), (+)-1-piresinolo (1.2%), and *p*-HPEA (0.6%) (Table 1).

**Figure 1:**
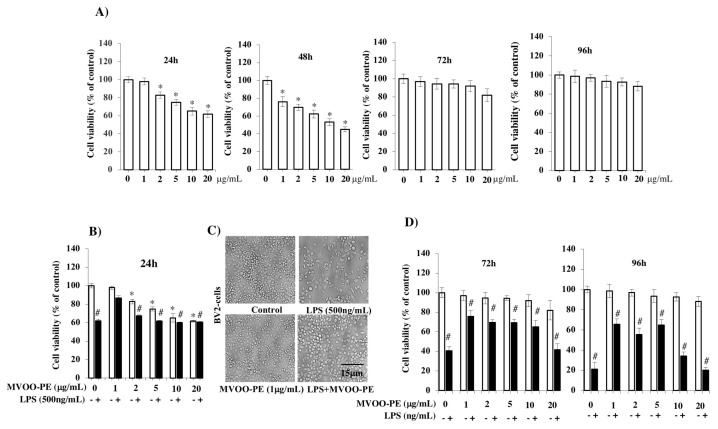
Effects of VOO phenolic extracts from Moraiolo cultivar (MVOO-PE) and LPS on BV2 microglia viability. (**A**) BV2 microglia cells were treated with the indicated concentration of MVOO-PE (1–20 μg/mL) for 24, 48, 72, and 96 h. Viability was tested using MTT test. (**B**) BV2 cells were pretreated with MVOO-PE (1 μg/mL) for 1 h before LPS (500 ng/mL) treatment. Cell viability was evaluated after 24 h using MTT assay. Data are mean ± standard deviation (SD) of three independent experiment performed in 8-fold. (**C**) microscopy analysis of BV2 microglia cells pretreated with MVOO-PE for 1 h before LPS treatment. Images were captured under bright field microscope with 20× magnification. Scale bar = 15 μm. (**D**) BV2 cells were pretreated with MVOO-PE 1 h before LPS treatment. Cell viability was evaluated after 72 and 96 h using MTT assay. Data are mean ± SD of three independent experiments performed in 8-fold. * *p* < 0.05 vs. untreated cells; # *p* < 0.05 vs. MVOO-PE-treated cells, according to the one-way ANOVA, followed by Fisher least-significant.

Cytotoxicity effects of the MVOO-PE towards BV2 cells were determined by measuring the cell viability using MTT assay after different incubation time: 24, 48, 72, and 96 h. As plotted in Figure 1A, concentrations from 1 to 20 μg/mL reduced progressively cell viability after 24 h of culture. The effect was more evident at 48 h when also 1 μg/mL concentration decreased cell viability. The cell proliferation rate was found not to be significantly reduced with concentration dependence at 72 and 96 h. This finding suggests that the cells present at the time of treatment had probably consumed all MVOO-PE, and they were less vital than control but yet they managed to divide. Thus, we set the cell viability study at 24 h in the LPS model.

In an attempt to select the most effective concentration of MVOO-PE for preventing the cytotoxicity induced by LPS, BV2 cells were treated with increasing concentrations of MVOO-PE (from 1 to 20 μg/mL) before incubation with 500 ng/mL LPS. Results highlighted that LPS alone reduced cell viability by 40% and that pretreatment with 1 μg/mL MVOO-PE was the most effective to significantly prevent cell death (Figure 1B). This effect was confirmed by microscopy analysis, where the combination of LPS + MVOO-PE appeared to prevent LPS-induced cell death (Figure 1C). This trend remained in time. In fact, LPS reduced by 60% and 80% cell viability at 72 h and 96 h, respectively. MVOO-PE prevented LPS toxicity and cell viability was recovered by about 26% at 72 h and 35% at 96 h (Figure 1D). Therefore, the protective efficacy of MVOO-PE on LPS-induced damage was not time-dependent, and we selected the 1 μg/mL concentration and the 24 h time point for subsequent experiments.

### 2.2. MVOO-PE Abolished Proinflammatory Cytokine Release and Inhibited the Activation of Inflammatory Mediators Induced by LPS Treatment

**Figure 2:**
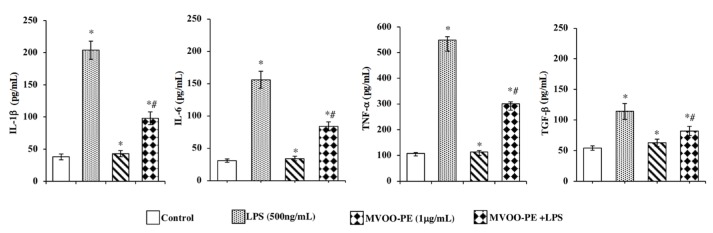
Effect of MVOO-PE on the production of IL-1β, IL-6, TNF-α, and TGF-β proinflammatory cytokines. BV2 cells were treated with LPS (500 ng/mL) or pretreated with MVOO-PE (1 μg/mL) for 1 h before LPS addition. The levels of cytokine secretion were measured in the cultured media after 24 h treatment. Data are expressed as picograms per milliliter pg/mL) and represent mean ± SD of three independent experiments. * *p* < 0.05 vs. untreated cells. # *p* < 0.05 vs. MVOO-PE-treated cells, according to the one-way ANOVA, followed by Fisher least-significant.

To evaluate the inhibitory effects of MVOO-PE on LPS-stimulated BV2 microglial cells, IL-1β, IL-6, TGF-β, and TNF-α levels in the cell culture media were measured by ELISA assay. The results clearly indicate that LPS alone was able to markedly induce an increase in IL-1β, IL-6, and TNF-α (5-fold increase) and TGF-β (2-fold increase) levels. Interestingly, a significant reduction of cytokine levels was observed following the incubation with MVOO-PE, indicating a clear protective action (Figure 2).

**Figure 3:**
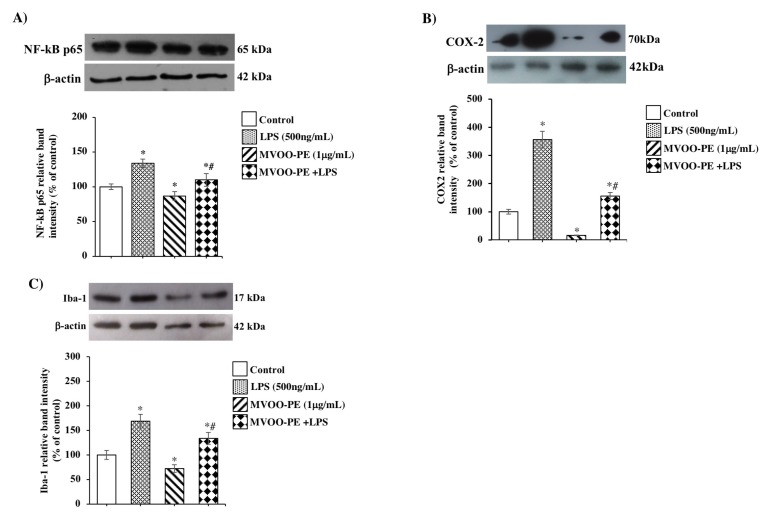
Effects of MVOO-PE on NF-kB–p65, COX-2, and Iba-1 inflammatory mediators. (**A**–**C**) BV2 microglia cells were treated with LPS (500 ng/mL) or pretreated with MVOO-PE (1 μg/mL) for 1 h before LPS addition. Western blot analysis was performed after 24 h treatment. Densitometry data of immunoblotting were normalized by β-actin levels and expressed as % of control. Data are mean ± SD of three independent experiments. * *p* < 0.05 vs. untreated cells. # *p* < 0.05 vs. MVOO-PE-treated cells, according to the one-way ANOVA followed by Fisher least-significant.

Many studies demonstrated the involvement of the TLR4–NLRP3 axis and NF-kB–p65 activation in neuroinflammatory response. Our results demonstrate that the treatment of BV2 cells with LPS induced NF-kB–p65 overexpression (Figure 3A) and, as a possible consequence, elevated levels of COX-2 and Iba-1 (Figure 3B,C). Higher levels were observed for COX-2 enzyme (a 3.5-fold increase), a key mediator of inflammatory pathways that results up-regulated in several diseases. Remarkably, MVOO-PE pretreatment significantly attenuated overactivation of COX-2 compared with LPS treatment alone, consistent with the notion that cytoprotective action occurs.

**Figure 4:**
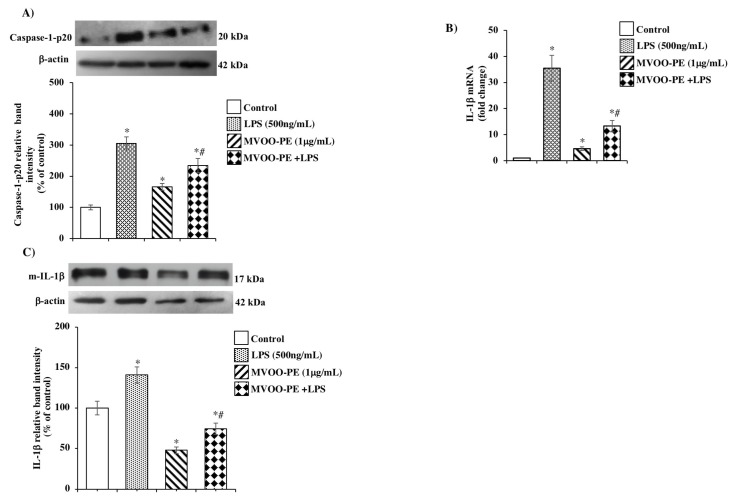
Effects of MVOO-PE on caspase-1 p20 and IL-1β expression. (**A**,**C**) BV2 microglia cells were treated with LPS (500 ng/mL) or pretreated with MVOO-PE (1 μg/mL) for 1 h before LPS addition. Western blot analysis was performed after 24 h. Densitometry data of immunoblotting were normalized by β-actin levels and expressed as % of control. Data represent mean ± SD of three independent experiments. (**B**) Gene expression analysis of IL-1β was performed by reverse-transcription real time PCR after 24 h treatment. Values were normalized to GAPDH expression and presented as 2^−ΔΔCT^. * *p* < 0.05 vs. untreated cells. # *p* < 0.05 vs. MVOO-PE-treated cells according to the one-way ANOVA followed by Fisher least-significant.

We then examined whether once primed, NLRP3 inflammasome assembly and consequent activation results in procaspase-1 cleavage and maturation in caspase-1 form and subsequently IL-1β release. Here, we found a significant caspase-1 up-regulation derived from LPS incubation (Figure 4A) and a strong increase in IL-1β mRNA (Figure 4B) and protein expression (Figure 4C). Interestingly, MVOO-PE pretreatment suppresses this cascade of processes, thus suggesting a key role of MVOO-PE in the attenuating the inflammatory status.

### 2.3. MVOO-PE Attenuated the TLR4 and NLRP3 Signaling Activation in LPS-Stimulated Microglial Cells

**Figure 5:**
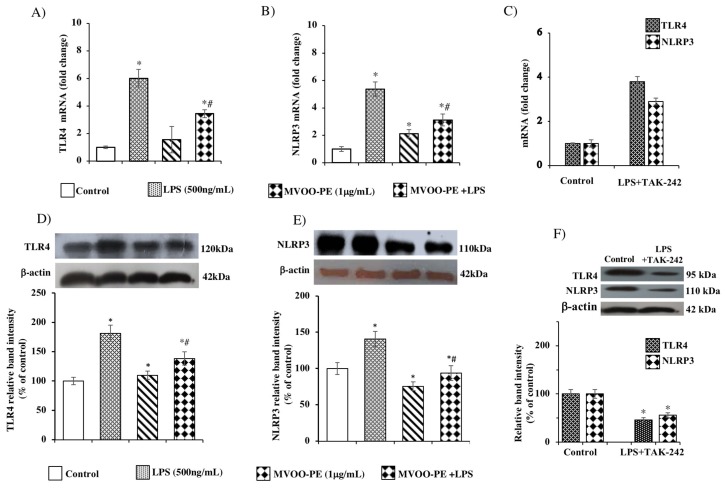
Effects of MVOO-PE on the TLR4/NLP3 axis. BV2 microglia cells were treated with LPS (500 ng/mL) or pretreated with MVOO-PE (1 μg/mL) for 1 h before LPS addition. TAK-242 (1 μM) was added 1 h before LPS treatment. Then, MVOO-PE was added. TAK-242 plus LPS and MVOO-PE remained in culture medium for the duration for the experiment (24 h). (**A**,**B**) Real-time PCR was performed to measure the TLR4 and NLRP3 mRNA levels. (**C**) mRNA levels of TLR4 and NLRP3 treated or not with LPS and TAK-242. GAPDH was used as housekeeping gene and values presented as 2^−ΔΔCT^. (**D**,**E**) Western blot analysis of TLR4 and NLRP3 protein levels treated or not with MVOO-PE and/or LPS. (**F**) Western blot analysis of TLR4 and NLRP3 protein levels treated or not with LPS plus TAK-242. Densitometry data of immunoblotting were normalized by β-tubulin levels and expressed as % of control. Data are mean ± SD of three independent experiments performed in triplicate. * *p* < 0.05 vs. untreated cells, according to the one-way ANOVA, followed by Fisher least-significant.

Many studies indicate that NLRP3 inflammasome acts as an important signaling molecule downstream TLR4. In order to investigate the respective role of TLR4 and NLRP3 in the protective action exerted by MVOO-PE, we first evaluated the impact of LPS on TLR4 and NLRP3, both on mRNA and protein levels. As expected, LPS exposure caused a marked increase of *TLR4* and *NLRP3* gene and protein levels (Figure 5A,B). Next, in order to establish whether NLRP3 was regulated by TLR4 activation [19], we used TLR4 specific signaling antagonist, TAK-242. Lower expression of *TLR4* gene and protein levels were observed when the cells were pretreated with TAK-242 (Figure 5C,F) with respect to LPS alone (Figure 5A,B), confirming the specific inhibition of TLR4 to LPS binding. This finding suggests that LPS up-regulates TLR4 by acting on its own receptor. Then, we tested the efficacy of MVOO-PE in modulating the TLR4/NLRP3 axis. We demonstrated, for the first time, that MVOO-PE pretreatment for 24 hat 1 μg/mL significantly reduced the LPS-evoked up-regulation of *TLR4* and *NLRP3* gene and protein expression (Figure 5D,E). These results confirm the engagement of the TLR4/NLRP3 axis for protective action of phenolic extract from olive oil.

### 2.4. MVOO-PE Prevented Neuronal Damage Induced by LPS Treatment

**Figure 6:**
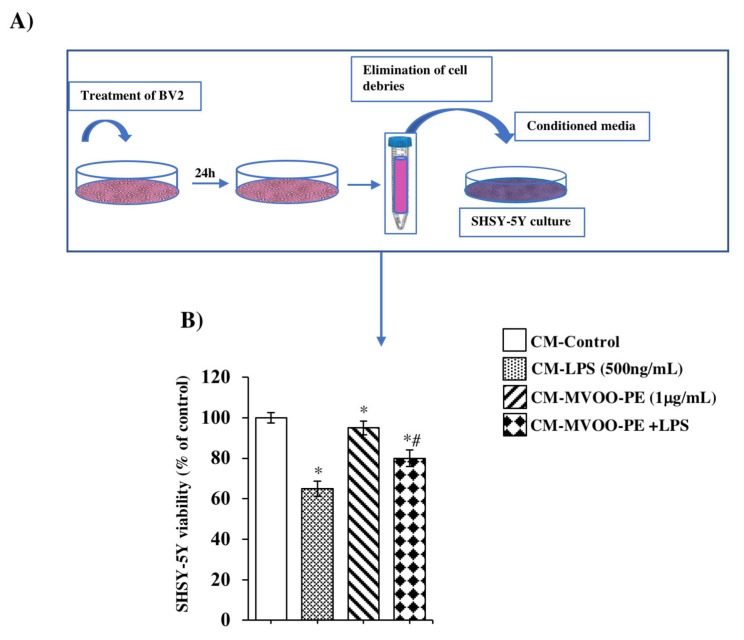
Effect of conditioned media from BV2 on SHSY-5Y viability. (**A**) Schema of conditioned media (CM) preparation (**B**) SHSY-5Y cell viability was assessed by MTT after treatment with CM for 24 h. Data are mean ± SD of three independent experiments performed in 8-fold. * *p* < 0.05 vs. untreated cells, according to the one-way ANOVA, followed by Fisher least-significant.

In order to examine the microglial–neuronal reciprocal influence following MVOO-PE preincubation, we generated an experimental model in which conditioned medium and co-culture system were used. First, we used conditioned media obtained from BV-2 cultures to stimulate SHSY-5Y neuroblastoma cell line, widely used in the neurodegeneration field. In Figure 6A, the experimental model for conditioned media is illustrated. Figure 6B showed that LPS-conditioned media reduced by 35% the SHSY-5Y cell viability, whereas only 20% cell viability decline was observed with LPS + MVOO-PE.

**Figure 7:**
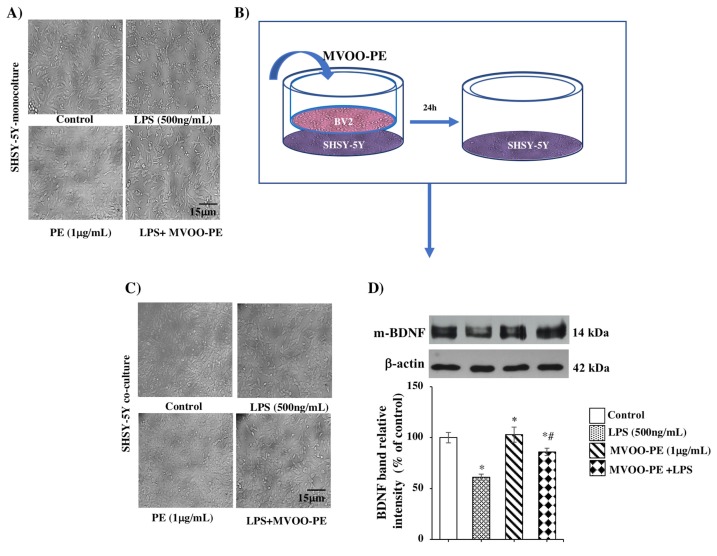
Effects of MVOO-PE on monoculture and microglia/neuron co-culture system. (**A**) SHSY5Y mono-cultures were incubated with MVOO-PE (1 μg/mL) before LPS addition (500 ng/mL), and 24 h later, microscopy images were captured. 20× magnification. Scale bar = 15 μm. (**B**) Microglia/neuron transwell co-culture system. (**C**) Images of SHSY-5Y were captured after 24 h of co-culture with BV2 cells by under bright field. 20× magnification. Scale bar = 15 μm. (**D**) Western blot analysis of BDNF protein levels. Densitometry data of immunoblotting were normalized by β-tubulin levels and expressed as % of control. Data represent mean ± SD of three independent experiments. * *p* < 0.05 vs. untreated cells. # *p* < 0.05 vs. MVOO-PE-treated cells, according to the one-way ANOVA, followed by Fisher least-significant.

Then, we directly stimulated SHSY-5Y with MVOO-PE prior to the addition of LPS. At 24 h incubation, microscopy analysis showed that 1 μg/mL MVOO-PE prevented neuronal damage induced by LPS (Figure 7A). Moreover, to investigate the interaction between microglia and SHSY-5Y neuron, we used a co-culture system in which BV2 microglia were grown on the permeable transwell membrane, and neurons were plated on the bottom of wells (Figure 7B). BV2 cells were stimulated as described above. Through microscopy analysis, we demonstrated that pretreatment with MVOO-PE protected SHSY-5Y against LPS injury (Figure 7C) and significantly prevented the reduction of BDNF (Figure 7D), a neurotrophic factor that plays an important role in neuronal survival and growth. These results suggest that this protection could be mediated by mutual influence between microglia and neuronal cells during MVOO-PE treatment.

## 3. Discussion

Virgin olive oil is universally recognized as a symbol of the Mediterranean diet. It is, at the same time, the main source of fat and the main health-promoting component with effects that include a reduced risk of cancer, neurodegenerative diseases [20], metabolic syndrome, and cardio-cerebrovascular events [21].

After food intake, olive oil phenolic compounds are adsorbed from the gastrointestinal tract and, through the systemic circulation, reach the brain. They are able to cross the blood brain barrier (BBB) endothelium exerting beneficial effects in different neuronal systems, although it is not yet completely clear whether the crossing occurs by simple diffusion or by specific carrier-mediated transport [22]. Nevertheless, the actual dose able to reach target tissues remains unexplored [23]

3,4-DHPEA and metabolites detected in human plasma are able to cross the BBB, interfering with amyloid aggregation, reducing oxidative stress, and regulating signaling pathways and proinflammatory cytokine secretion [24]. In addition, the brain uptake and accumulation of hydroxytyrosol and its metabolites were observed after 21 days of rat diet supplementation, and their neuroprotective potential was observed [25].

The concentration of polyphenols in MVOO is affected by many different factors, such as olive cultivar, geographical area, age of the tree, agronomic and environmental factors, degree of ripeness as well as by the extraction system and storage conditions.

The present study was aimed at investigating the anti-inflammatory efficacy of phenolic extracts derived from Moraiolo cultivar in an in vitro model of neuroinflammation.

We examined the action of MVOO-PE on BV2 viability and different proinflammatory mediators’ release and expression. Then, we explored the involvement of TLR4/NLRP3 axis and downstream IL-1β processing, through the activation of caspase-1.

Although the importance of consuming foods rich in phenolic compounds has been highlighted by other authors, we think that the novelty of this paper is to consider the synergistic effect of all the phenolic compounds, rather than the activity from single molecules. The phenolic profile of virgin olive oil is unique for the exclusive presence of secoiridoid derivatives (3,4-DHPEA-EDA, 3,4-DHPEA-EA, *p*-HPEA-EDA, and ligstroside aglycon), not occurring in other edible fruit derivatives, other than phenolic alcohols such as hydroxytyrosol and tyrosol.

The high antioxidant activity of 3,4-DHPEA, 3,4-DHPEA-EDA, and 3,4-DHPEA-EA has been demonstrated. Secoiridoids have been recognized for their ability to inhibit blood platelet aggregation and thromboxane synthesis in human cells, as well as inhibition of phospholipids and LDL oxidation. Protection of human erythrocytes from the oxidative damage was also shown [26]. A diet rich in olive oil is believed to confer health benefits that overlap with those attributed to nonsteroidal anti-inflammatory drugs. Long-term consumption of p-HPEA-EDA may help to protect against some diseases by virtue of its ibuprofen-like COX-inhibiting activity [27]. Abuznait and collaborators showed the in vitro and in vivo effects of p-HPEA-EDA in reducing the accumulation of amyloid plaques and enhancing β-amyloid clearance from the BBB [28]. Moreover, a human in vivo study revealed the ability of olive oil phenolic compounds to modulate the expression of atherosclerosis-related genes [29]

In the CNS, microglia become readily active in response to brain injury or immunological stimuli to promote host defense by destroying invading pathogens. Upon activation, microglia also promotes tissue repair and homeostasis, by affecting surrounding astrocyte and neurons. However, long-term over-activation of microglia can lead to excessive production of inflammatory mediators and neuronal injury. Increasing evidence suggests that signaling pathways downstream TLR4 play an important role in the pathogenesis of neuroinflammation. TLR4 is a pattern recognition receptor that activates a cascade of events in response to LPS endotoxin binding, culminating in over-production of inflammatory mediators and cytokines.

Thus, the pharmacological inhibition of binding of LPS to TLR4 represents an intriguing therapeutic strategy for treatment of neuroinflammatory diseases.

Glial cells also express PRRs, such as NOD-like receptors (NLRs) [10], recognized as downstream mediators of inflammasome able to sense a plethora of stimuli [30] Among NLR inflammasome complexes, the NLRP3 has been the most widely characterized [11]. The over-production of IL-1β, IL-6, and TNF-α cytokines is a hallmark of neurological diseases in the brain. Therefore, the selective attenuation of cytokine secretion may control neuro-inflammatory disorders. TGF-β can exert both pro- and anti-inflammatory activity depending on the activation state of the cell. It is capable of converting an active site of inflammation into one dominated by resolution and repair [31].

Here, we highlighted the anti-inflammatory effects of MVOO-PE against LPS-induced cytotoxicity and consequent release of IL-1β, IL-6, TNF-α, and TGF-β proinflammatory cytokines. We provided evidence for the mechanism involved in the downstream inflammatory response, pointing to the TLR4–NLRP3 axis.

Upon activation, NLRP3 inflammasome induces the maturation of pro-caspase to caspase-1 that provide the cleavage of pro-IL-1β into biologically active IL-1β. We found that LPS caused the overexpression of TLR4 and NLRP3, probably through NFkB–p65 activation, and consequent up-regulation of COX2 and Iba-1 inflammatory mediators. Moreover, we found increase of caspase-1 protein levels, undoubtedly to provide cleavage of IL-1β cytokine and to produce IL-1β mature form. Interestingly, the increase in *TLR4*, *NLRP3*, and *IL-1β* gene and protein expression were suppressed by MVOO-PE treatment. These findings support the notion that TLR4/NLRP3 regulation was essential for protection of microglia cells. By using TAK-242 specific inhibitor, the involvement of TLR4 engagement was confirmed.

In the brain, highly relevant are the interactions between neurons and microglia. Several soluble molecules secreted by either microglial or neuronal cells have been implicated in the mutual influence between these cells. Considering this crosstalk, we further investigated the role of MVOO-PE on neuronal survival by using conditioned media and a microglia/neuron co-culture system.

**Figure 8:**
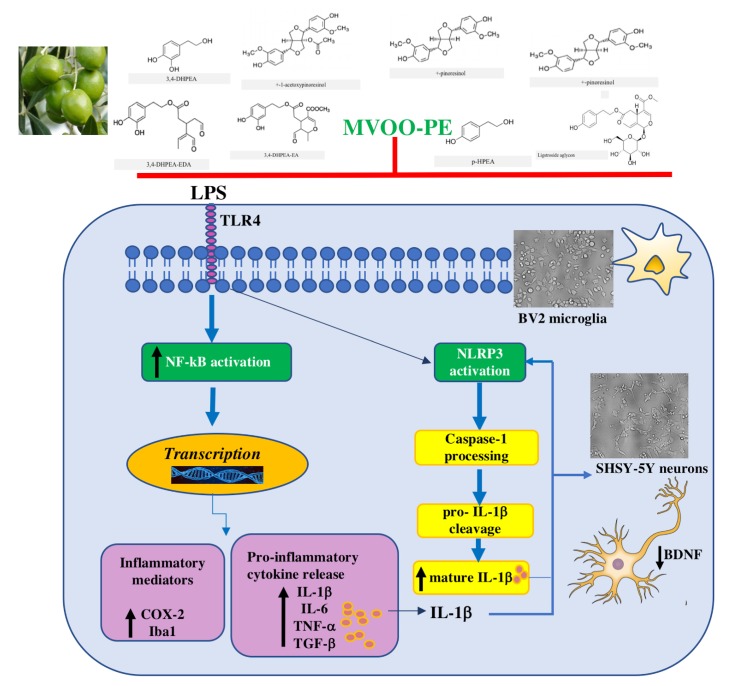
Proposed mechanism of anti-inflammatory effects by MVOO-PE on microglia/neurons. MVOO-PE prevents the LPS-induced signaling cascade downstream TLR4 in BV2 cells. Specifically, MVOO-PE abolished the overactivation of NF-kB and consequent up-regulation of COX-2 and Iba-1 inflammatory mediators. The increase of proinflammatory cytokines such as IL-1b, IL-6, TNF-α, and TGF-β was also counteracted. The activation of NLRP3 is responsible for caspase-1 processing of pro-IL-1b to mature IL-1b, which in turn further activates NLRP3, causing the impairment of adjacent neurons and reduction of BDNF levels. Pretreatment with MVOO-PE is able to prevents all of these harmful events (red bar).

We showed that incubation of SH-SY5Y neurons with conditioned media derived from BV2 treated with MVOO-PE plus LPS caused a slight but significant increase of cell viability compared to conditioned media from LPS alone. Finally, microscopy analysis of SHSY-5Y from co-culture showed a strong neuroprotective effect derived from MVOO-PE pretreatment, as well as a slight but significant attenuation of BDNF decrease. As BDNF represents the most active neurotrophin/growth factor against neuroinflammation, our findings could suggest that MVOO-PE regulates inflammatory response also by preserving BDNF levels. The results of this study are schematically shown in Figure 8.

## 4. Materials and Methods

### 4.1. Chemicals

Hydroxytyrosol (3,4-DHPEA) and tyrosol (*p*-HPEA) were supplied, respectively, by Fluka (Milan, Italy) and Cabru s.a.s. (Arcore, Milan, Italy), whereas the dialdehydic forms of elenolic acid linked to 3,4-DHPEA and *p*-HPEA (3,4-DHPEA-EDA and *p*-HPEA-EDA), the isomer of oleuropein aglycon (3,4-DHPEA-EA), ligstroside aglycon, and lignans ((+)-1-acetoxypinoresinol and (+)-pinoresinol) were obtained as described by Montedoro et al. [32] and Servili et al. [33].

### 4.2. Extraction of Virgin Olive Oil Phenolic Extracts (VOO-PE)

Virgin olive oil was obtained by a mechanical extraction process performed as follows. Green olives from cultivar Moraiolo (*Olea europaea* L.) were crushed using a hammer crusher; malaxation was carried out for 40 min at 25 °C, and the oil was separated by centrifugation (9600× *g*, 1 min) using a decanter at a low level of water addition. The virgin olive oil phenolic extract (VOO-PE) was obtained according to previously published method [34].

### 4.3. VOO-PE HPLC Analysis

The HPLC analysis of phenolic compounds of EVOOs was carried out using Agilent Technologies system, model 1100 (vacuum degasser, quaternary pump, autosampler, thermostated column compartment, diode array detector (DAD), fluorescence detector (FLD)) controlled by ChemStation (Agilent Technologies, Palo Alto, CA, USA) to evaluate the chromatographic data as described by Selvaggini et al. [35]. Phenolic compounds were evaluated using a Spherisorb ODS-1 250 × 4.6 mm column with a particle size of 5 μm (Waters, Milford, MA, USA). The mobile phase consisted of 0.2% acetic acid (pH 3.1) in water (solvent A)/methanol (solvent B) at a flow rate of 1 mL/min. The gradient changed as follows: 95% A for 2 min, 75% A in 8 min, 60% A in 10 min, 50% A in 16 min, and 0% A in 14 min and was maintained for 10 min; the total running time was 73 min. All phenolic compounds were detected by DAD at 278 nm, with the only exception of lignans detected by FLD, activated at an excitation wavelength of 280 nm and emission at 339 nm.

### 4.4. Cell Cultures

Microglial BV-2 cell line was cultured in RPMI-1640 medium (EuroClone, Pero, MI, Italy), supplemented with 0.1% penicillin–streptomycin and 5% defined fetal bovine serum (D-FBS; HyClone™ U.S.). Cells were maintained at 37 °C in a 5% CO_2_ incubator. Confluent cultures were passed every 2–3 days.

SH-SY5Y neuroblastoma cells were maintained at 37 °C in 5% CO_2_ in RPMI-1640 medium (EuroClone, Pero, MI, Italy), supplemented with 0.1% penicillin–streptomycin and 10% FBS. Compounds were supplied as ethanolic solution and stored at −20 °C in the dark. The mixture was diluted in RPMI 1640 just before use. All the solutions were sterilized by filtration on 0.22 μm filters (EuroClone, Pero, MI, Italy).

### 4.5. Experimental Design

VOO phenolic extracts from Moraiolo cultivar (MVOO-PE) were dissolved as a stock solution (5 mg/mL) in pure ethanol. BV-2 cells were treated with increasing concentrations of MVOO phenolic extract diluted with RPMI-1640 medium (EuroClone, Pero, MI, Italy) supplemented with 5% D-FBS to 1, 2, 5, 10, and 20 μg/mL final concentration. After 1 h, the cells were stimulated with LPS (500 ng/mL) (*Escherichia coli*, stock solution 5 mg/mL) and incubated for different exposure times, such as 4, 24, 48, 72, and 96 h. To test the TLR4 involvement, cells were pre-treated with TAK-242 (1 μM) for 1 h, prior to treatment with LPS or VOO-PE. TAK-242 remained in culture medium for the duration for the experiment.

### 4.6. Preparation of Conditioned Media

Conditioned media were obtained from BV-2 microglia cultures as described by Baroni et al. [36] Briefly, BV-2 cells were seeded in 24 cm^2^ flasks in RPMI-1640 (EuroClone, Milano, Italy), supplemented with 0.1% penicillin–streptomycin and 5% D-FBS, and treated with MVOO phenolic extract (1 μg/mL). After 1 h, the cells were stimulated with LPS (500 ng/mL) and incubated for a further 24 h. Then, media were collected and centrifuged to eliminate any cellular debris and stored at −80 °C until use. Conditioned media (CM) were used to test SH-SY5Y viability by MTT.

### 4.7. Co-Culture of Neurons and Microglia

A transwell co-culture device with two chambers separated by a semi-permeable 0.4 µm membrane (Transwell plate, Corning Incorporated, New York, NY, USA,) was used to investigate the mutual influence between microglia and SHSY-5Y dopaminergic cell line. Briefly, SHSY-5Y cells (2 × 10^5^ cells/mL) were seeded onto 6-well plates (lower chamber) in RPMI supplemented with 10% FBS and incubated at 37 °C in a 5% CO_2_ incubator. Twenty-four hours later, BV-2 cells (2 × 10^5^ cells/mL) were seeded onto the upper insert wells in RPMI supplemented with 5% D-FBS and incubated for 24 h. Then, culture media of the upper and lower inserts were replaced with fresh serum-free RPMI. MVOO-PE (1 μg/mL) was added to BV2 1 h before LPS (500 ng/mL) treatment. Finally, the SH-SY5Y cells in the lower plate wells were harvested using scraper and collected for the experiments.

### 4.8. MTT Assay

Cell viability was determined by the conventional 3-(4,5-dimethylthiazol-2-yl)-2,5-diphenyltetrazolium bromide (MTT) reduction assay, as described by Minelli et al. [37], with some modifications. BV-2 microglia cells (4 × 10^4^ cells/well) were plated into 96-well plates. After treatments, 10 μL of MTT (stock solution 5 mg/mL) per well was added and incubated for 4 h at 37 °C. Finally, the formazan crystals were dissolved in 100 μL DMSO and the absorbance of each well was recorded at 595 nm using a plate reader. The percent viability was calculated as optical density (OD) of the treated sample/control OD × 100. For all experiments, 1 μg MVOO-PE concentration was selected.

### 4.9. Real-Time PCR (RT-PCR)

Total RNA was isolated with an RNeasy Mini Kit (Qiagen, Milano, Italy) and a Direct-zol RNA MiniPrep Kit (Qiagen, Milano, Italy). RNA concentration was determined with spectrophotometry. First, 1 μg of RNA was used to synthetize cDNA with a Revertaid Premium First Strand cDNA Synthesis Kit (Thermo Fisher Scientific, Waltham, MA, USA). Then, the gene expression was detected by real-time PCR (RT-PCR), using SYBR Green Master Mix (Roche, Monza, Italy) as previously described [38]. Data were obtained and elaborated with iCyclerQ detection system (Roche, Monza, Italy). Primer sequences (Thema Ricerca, Bologna, Italy) are listed in Table 2.

### 4.10. Western BLOT Analysis

BV-2 cells were detached with scraper, centrifuged for 7 min at 1500 rpm, and washed twice with cold PBS. After centrifugation, pellets were recovered and 200 μl of RIPA lysis extraction buffer was added (Thermo Fisher Scientific, Waltham, MA, USA). The samples were put on ice for 30 min, shaking every 5 min. The cell lysate was sonicated (10 sec, 3 cycles, with 10 sec intervals with high intensity). After that, samples were centrifuged (10 min at 13,000 rpm) and supernatants were collected and stored at −80 °C until use. Protein concentration was determined by the BCA method and analyzed as previously reported [39,40]. Then, 60 μg of denatured (100 °C for 10 min) total protein extracts was subjected to SDS-PAGE on a 10 or 15% polyacrylamide gel, together with a molecular weight marker (Prestained Protein SharpmassVI Protein MW marker, 5–245 kDa (EuroClone, Milano, Italy)), transferred to a nitrocellulose membrane and blocked with 5% milk in TBS (Tris 50 mM, NaCl 150 mM, Tween 20%, pH 7.4) for 1 h at room temperature. The membranes were incubated overnight at 4 °C with the following primary monoclonal antibodies: Mouse anti-TLR4 (1:1000, Cell Signaling Technology, Danvers, MA, USA), rabbit anti-NLRP3 (1:2000, Cell Signaling Technology, Danvers, MA, USA), rabbit anti-NF-kBp65 (1:1000, Cell Signaling Technology, Danvers, MA, USA), mouse anti-caspase-1 (1:500, Abcam, Cambridge, UK), mouse anti-Iba1 (1:500, Santa Cruz Biotechnology, Inc., Santa Cruz, CA, USA), mouse anti-COX2 (1:1000, Santa Cruz Biotechnology, Inc.), and Armenian hamster anti-IL-1β, rabbit anti BDNF (1:1000, Santa Cruz Biotechnology, Inc.). β-actin mouse monoclonal antibody was used as loading control (1:2000, Santa Cruz, Biotechnology, Inc.). All antibodies were diluted in PBS with 0.1% Tween-20 (T-PBS). Then, the membranes were washed in T-PBS and incubated in horseradish peroxidase (HRP)-labeled secondary antibodies for 2 h at room temperature: Anti-rabbit for NLRP3, NF-kBp65, and BDNF (1:1000, Cell Signaling Technology, Danvers, MA, USA), anti-mouse for β-actin, TLR4, Iba1, Caspase, 1, COX2 (1:10,000, Cell Signaling Technology, Danvers, MA, USA), and anti-Armenian hamster for IL-1β (1:5000, Santa Cruz Biotechnology, Inc.) were used. The protein bands were visualized using an ECL detection kit (Thermo Fisher Scientific, MA, USA), and blot images were captured and analyzed by ImageJ ProPlus software (version 1.51i, National Institute of Mental Health, Bethesda, MA, USA).

### 4.11. Measurement of Cytokine Levels

After incubation period, BV-2 cells culture media were replaced with fresh serum-free RPMI-1640 and cells incubated for a further 24 h. Media were collected, centrifuged to eliminate cell debris, and stored at −80 °C until use. IL-1β, IL-6, TNF-α, and TGF-β cytokines were measured by commercial enzyme-linked immunosorbent assay (ELISA) kits (Thermo Fisher Scientific), according to the manufacturer’s instructions.

### 4.12. Statistical Analyses

Data are expressed as means ± standard deviation (SD). Differences among groups were evaluated by one-way analysis of variance (ANOVA), followed by Fisher least-significant difference to test the means that were significantly different from the control means. *p*-values <0.05 were considered to be statistically significant.

## 5. Conclusions

In conclusion, all of the findings of the present study supported the evidence for the anti-inflammatory and neuroprotective efficacy of olive oil phenolic extracts obtained from Moraiolo cultivar. Taken together, the results support the hypothesis for beneficial efficacy of virgin olive oil through the modulation of TLR4/NLRP3 signaling. Targeting this axis may represent a promising approach for prevention of several neuroinflammatory diseases.

## Figures and Tables

**Table 1:** Phenolic compound contained in Moraiolo cultivar olive oil (MVOO).

	Phenolic Compounds (mg/g)
3,4-DHPEA-EDA	359.3 ± 11.3
3,4-DHPEA-EA	189.0 ± 3.3
*p*-HPEA-EDA	107.5 ± 0.4
(+)-1-Acetoxypinoresinol	35.8 ± 0.1
Ligstroside aglycone	17.7 ± 0.4
3,4-DHPEA	15.2 ± 0.1
(+)-Pinoresinol	8.7 ± 0.1
*p*-HPEA	4.3 ± 0.001
Total polyphenols	737.5 ± 11.8

**Table 2:** List of primer sequences used for RT-PCR.

**Accession Number**	**Gene Symbol**	**Primer Sequences (F: Forward; R: Reverse)**
**NM_007393**	*β-act*	F. AGA GGG AAA TCG TGC GTG AC R. CAA TAG TGA TGA CCT GGC CGT
**NM_019467**	*Iba1*	F. GGATTTGCAGGGAGGAAAAG R. TGGGATCATCGAGGAATTG
**NM_008361**	*IL-1β*	F. AAGGAGAACCAAGCAACGACAAAA R. TGGGGAACTCTGCAGACTCAAACT
**NM_145827**	*NLRP3*	F. AAAATGCCTTGGGAGACTCA R. AAGTAAGGCCGGAATTCACC
**NM_021297**	*TLR4*	F. TTCACCTCTGCCTTCACTAC R. CACTACCACAATAACCTTCCG
